# Identification of new fluorophores in coelomic fluid of *Eisenia andrei* earthworms

**DOI:** 10.1371/journal.pone.0214757

**Published:** 2019-03-28

**Authors:** Jerzy Kruk, Michał Dziurka, Barbara Płytycz

**Affiliations:** 1 Department of Plant Physiology and Biochemistry, Faculty of Biochemistry, Biophysics and Biotechnology, Jagiellonian University, Gronostajowa, Kraków, Poland; 2 Polish Academy of Sciences, Institute of Plant Physiology, Niezapominajek, Kraków, Poland; 3 Department of Evolutionary Immunology, Institute of Zoology and Biochemical Research, Faculty of Biology, Jagiellonian University, Gronostajowa, Kraków, Poland; Imperial College London, UNITED KINGDOM

## Abstract

Coelomic fluid of *Eisenia andrei* contains a number of UV-fluorescent compounds. In the present study we have found that four of these compounds showed identical fluorescence excitation and emission maxima at 310 nm and 364 nm, respectively, suggesting they share the same chromophore. NMR and HR-MS spectroscopy of the most abundant fluorophore reavealed that its molecule is composed of two quinazoline-2,4-dione rings connected by spermine linker. This compound was earlier indentified in *Eisenia andrei* as SP-8203. Moreover, we have identified the structure of the two other fluorophores, one differing from SP-8203 by the absence of N-acetyl group, the compound not reported in any other organisms before, and the other already found in *E*. *fetida* and regarded as species specific. However, our results indicate that this metabolite is also present in *E*. *andrei* in significant amounts. The possible origin and function of these new metabolites is discussed.

## Introduction

Morphologically similar lumbricid species occupying the same ecological niche, *Eisenia andrei* and *E*. *fetida*, can be phenotypically differentiated by metabolic profiling of tissue extracts and/or coelomic fluid [1]. Fluorescence spectra of coelomic fluid of these species were for the first time used for taxonomic purposes by Albani et al. [2], and *E*. *andrei* specific fluorophore was suggested as 4-methylumbelliferyl β-D-glucoronide (MUGlcU). In fact, similar spectra have been shown for methanol solutions of 4-methyl umbelliferone, a member of coumarin family [3].

Specific fluorescence spectra with a peak of excitation at 310–320 nm and a peak of emission at 370–380 nm, were used by our group as one of taxonomic markers of *E*. *andrei*, and called the MUG, MUG-like, or the M fluorescence. However, during these studies the MUG/MUG-like fluorescence spectra were detected not only in *E*. *andrei* but also in a few of *E*. *fetida* earthworms [4–6] that inspired us for studies on hybridisation between laboratory joined inter-specific pairs of M-positive *E*. *andrei* with M-negative *E*. *fetida*. The results revealed the existence of asymmetrical hybridization between these hermaphroditic species able to self-fertilization [7], with hypothetical M fluorophore inherited by *E*. *andrei*-derived hybrids and then transferred to some of their *E*. *fetida* offspring [8]. Recently, interspecific hybrids between *E*. *andrei* and *E*. *fetida* have been found among field-samples earthworms from Scandinavian populations [9]. Perhaps the hypothetical M-fluorophore might be one of the markers of interspecific gene flow, thus studies on its proper identification and characteristics are pertinent.

The fluorescent properties of *E*. *andrei* fluorophore are evidently different from that of 4-methylumbelliferyl-β-D-glucuronide [10]. Moreover, its fluorescence is not affected by β-glucuronidase (Kruk, unpublished) in contrast to MUG [10].

An aromatic metabolite, called SP-8203, was isolated from coelomic fluid of *E*. *andrei*, consisting two quinazoline-2,4-diones joined by an N-acetylspermine linker [11, 12]. Later, its presence in coelomic fluid of *E*. *andrei* has been confirmed [13], although in none of these studies its fluorescence properties were analyzed.

In the present research we decided to isolate the most abundant fluorophores of *E*. *andrei* and determine their molecular structure and spectral properties.

## Materials and methods

Investigations were performed on progeny of the composting earthworm species *Eisenia andrei* received from the laboratory stocks of the University in Lille (France) and reared for generations in the laboratory of Institute of Zoology and Biomedical Research of the Jagiellonian University (Krakow, Poland). In Krakow worms were routinely kept in boxes with commercial soil at 17°C, 12:12 LD, and fed with a mixed diet comprised of dried/boiled nettle (*Urtica dioica*) and dandelion leaves (*Taraxacum officinalis*), and boiled/dried tea leaves. Adult (clitellate) earthworms of similar body weights were used for present investigations.

One hundred of adult individuals of *E*. *andrei* (total weight 30.4 g) was divided into 4 groups (25 individuals each, biomass ca. 7.5 g). Each group was immersed in 10 ml of PBS (Fresenius Kabi Poland, Warsaw) and shocked for 60 s with 4.8V constant current. The solutions were pooled and treated twice by freeze-thaw cycles in liquid nitrogen. After centrifugation (5000*g* x 10 min) the sediment was discarded, while the supernatants were lyophilized and finally dissolved in 10 ml of the HPLC solvent (0.1% formic acid/ acetonitrile, 82.5/17.5 v/v).

Fluoresce spectra were recorded on a Perkin Elmer LS55 spectrofluorimeter using 5/5 nm excitation/emission slits, using quartz cuvette with 10 mm optical pathway.

N-butyl-2,4-quinazolinedione was synthesized from anthranilic acid and *n*-butylcarbamate (both from Sigma-Aldrich) according to the method of Michman et al. [14].

### HPLC measurements

Semipreparative HPLC was performed using Acclaim C30 RP column (4.6 x 250 mm, 5 μm) in 0.1% formic acid/ acetonitrile, (82.5/17.5, v/v) (isocratic mode) at the flow rate 1.2 ml/min and 500 μl injection loop. During chromatography, tandem detection of absorption at 320 nm and fluorescence at 320/390 nm excitation/emission was monitored. The setup included Jasco PU-2080 Plus pump, UV-970 UV/VIS absorption detector and Shimadzu RF-10 AXL fluorescence detector.

### NMR measurements

NMR spectra were measured using Agilent DD2 600 MHz and Bruker Avance III HD 800 MHz instrument eqiupped with the cryogenic TCI probe in H_2_O/D_2_O mixture (9/1, v/v). Experimental details: ^1^H 600 MHz Agilent DD2—pulse program:water. Parameters:sw = 12 kHz, at = 1 s np = 24038 zero filing 65536, nt = 16, LB = 0, d1 = 1.5 s. ^13^C 800 MHz Bruker Avance IIIHD—pulse program: zgpg30. Parameters:sw = 48.08 kHz, AQ = 1.5 s TD = 144224 zerofiling 262144, NS = 10000, LB = 1, d1 = 2s. ^1^H decoupling performed with waltz16. COSY800 MHz—pulse program: cosygpprqf. Parameters:sw: 7995.7x 7995.7 Hz,ns = 4; ds = 16; d1 = 1.5s1024x1024 complex points; zerofiling 2048x2048, sinebellapodization, absolute value spectrum. HSQC800 MHz—pulse program: chsqcbb. Parameters: sw: (^1^H) 12.82 kHz x (^13^C)33.18 kHz, ns = 8; ds = 16; d1 = 1.5s, 1024x2048 complex points, zero filing 2048x4096, cosinebellapodization. HMBC 800 MHz—pulse program: hmbcgplpndprqf. Parameters: sw: (^1^H) 10.42 kHz x (^13^C) 44.24 kHz, ns = 32; ds = 16; d1 = 1.5 s, 512x512 complex points, zero filing 1024x1024, sinebellapodization, absolute value spectrum. All spectra were referenced according to IUPAC guidelines (*Pure Appl*. *Chem*., Vol. 73, No. 11, pp. 1795–1818, 2001), no internal standard was used.

### LR-MS measurements

Low resolution mass spectra were recorded after HPLC separation on quadrupole tandem mass spectrometer (HPLC-QQQ MS/MS, 6410 LC/MS, Agilent, Santa Clara, CA, USA). The ion source was electro-spray ionization in positive mode (+ESI), at capillary voltage 4 kV, gas temperature 350°C, gas flow 12 L/min and nebuliser pressure 35 psi. Separation was performed on AscentisExpress C18 (3×150 mm, 2.7 μm; Supelco, Bellefonte, PA, USA) analytical column in isocratic mode at 0.5 ml/min, injection was 5 μl, the same solvent mixture was used as for semipreparative separation.

### HR-MS measurements

Mass spectrometry analyses were performed using Synapt G2-S mass spectrometer (Waters) equipped with the electrospray ion source and quadrupole-Time-of-flight mass analyzer. Sample was dissolved in methanol and injected directly into the electrospray ion source.Methanol was used as a solvent with the flow rate 100 μl/min. The measurement was performed in positive ion mode with the resolving power of the TOF analyzer 20000 FWHM. The instrument worked with external calibration on sodium formate in the mass range of *m/z =* 50–2000. The lock spray spectrum of the leucine-enkephalin was generated by the lock spray source and correction was done for every spectrum. The exact mass measurements for all peaks were performed within 3 mDa mass error. The nitrogen was used as desolvation and cone gas and their flow values were set to 861 L/h and of 222 L/h respectively.The desolvation gas flow was and temparature 150°C. The nebulizer gas pressure was set to 5.8 bar. The capillary voltage was set to 3.8 kV, and the sampling cone voltage and source offset were set to 20 V. The instrument was controlled and recorded data were processed using the MassLynx V4.1 software package (Waters). The quantity of compound 6, used for NMR studies was 7 mg.

## Results

**Fig 1:**
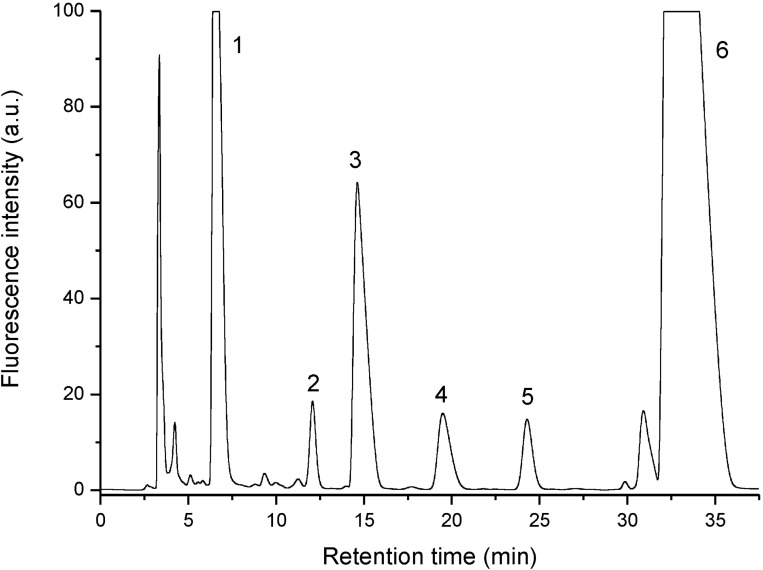
HPLC chromatogram of preparative purification of UV-fluorescent compounds of *E*. *andrei* coelomic fluid. Fluorescence was followed using 320/390 nm excitation/emission.

**Fig 2:**
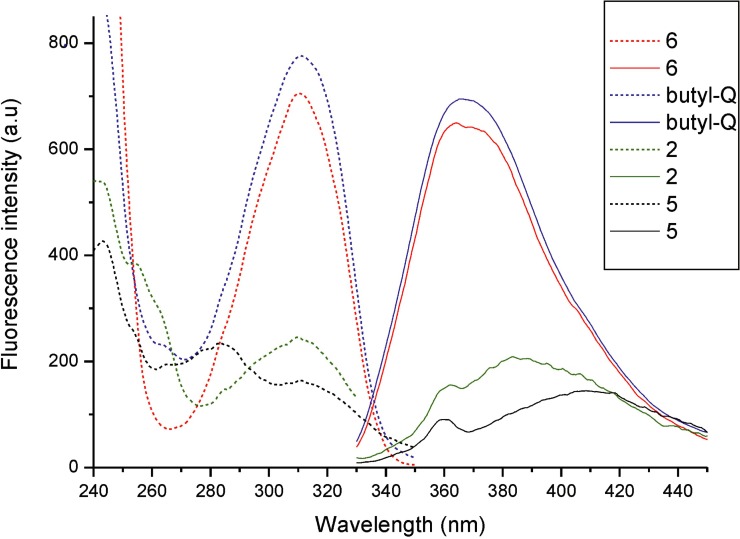
Excitation and emission spectra of *E*. *andrei* UV-emitting fluorophores and of *n*-butyl-quinazolinedione. Excitation was at 320 nm for emission spectra, while emission was set at 390 nm for excitation spectra. The peak at 360 nm for compounds 2 and 5 in the emission spectra originates from Raman scattering.

**Fig 3:**
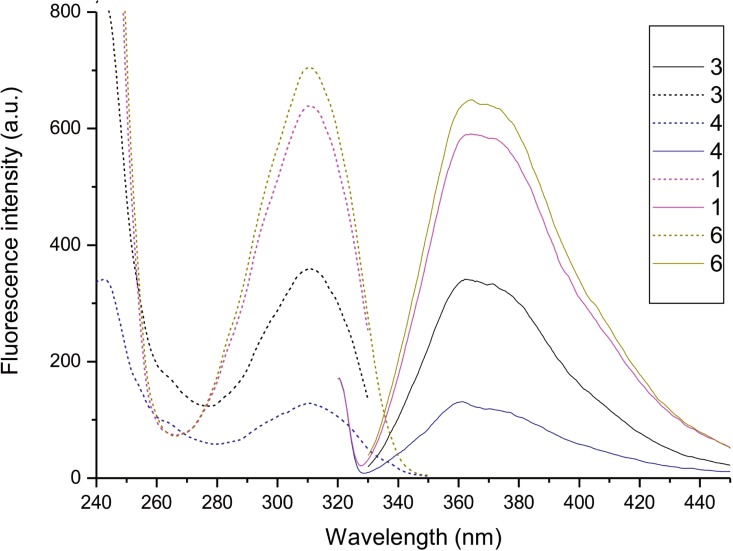
Excitation and emission spectra of fluorophores 1, 3, 4 and 6. Excitation was at 320 nm for emission spectra, while emission was set at 390 nm for excitation spectra.

The HPLC chromatogram of coelomic fluid extract showed (Fig 1) that it contains a mixture of several fluorescent compounds, however, the fluorophore corresponding to peak 6 is predominant. The percent composition of the fluorophores was as follows: 11.9% (compound 1), 0.7% (2), 9.0% (3), 2.6% (4), 0.6% (5) and 75.2% (6). The spectral data of the fluorophores (Table 1, Figs 2 and 3) show that the compounds corresponding to peaks 1, 3, 4 and 6 in the chromatogram show the same excitation and emission maxima, suggesting that they have the same chromophore. For compounds 2 and 5, the emission maxima are at longer wavelengths but have the same excitation maxima at 310 nm (Table 1, Fig 2). This indicates that chromophores of these compounds are of different but similar structure to those of compounds 1, 3, 4 and 6. As minor components, the compounds 2, 4 and 5 were not further analyzed in the present studies.

**Table 1:** Excitation and emission maxima of *E*. *andrei* UV-emitting fluorophores and of synthetic *n*-butyl-quinazolinedione (butyl-Q).

Peak number/compound	Excitation maximum (nm)	Emission maximum (nm)
1	310	364, 370 sh.
2	310	385
3	310	364, 370 sh.
4	310	364, 370 sh.
5	284, 310 sh.	410
6	310	364, 370 sh.
butyl-Q	310	365, 370 sh.

Fluorescence spectra were measured in the HPLC solvent (0.1% formic acid/acetonitrile, 82.5/17.5 v/v), sh–shoulder. The purity of compound 6 was 96%, while that of butyl-Q—83%. The milimolar extinction coefficient in methanol for compound 6 was ε = 4.80 and for butyl-Q it was ε = 2.07 at 310 nm.

**Fig 4:**
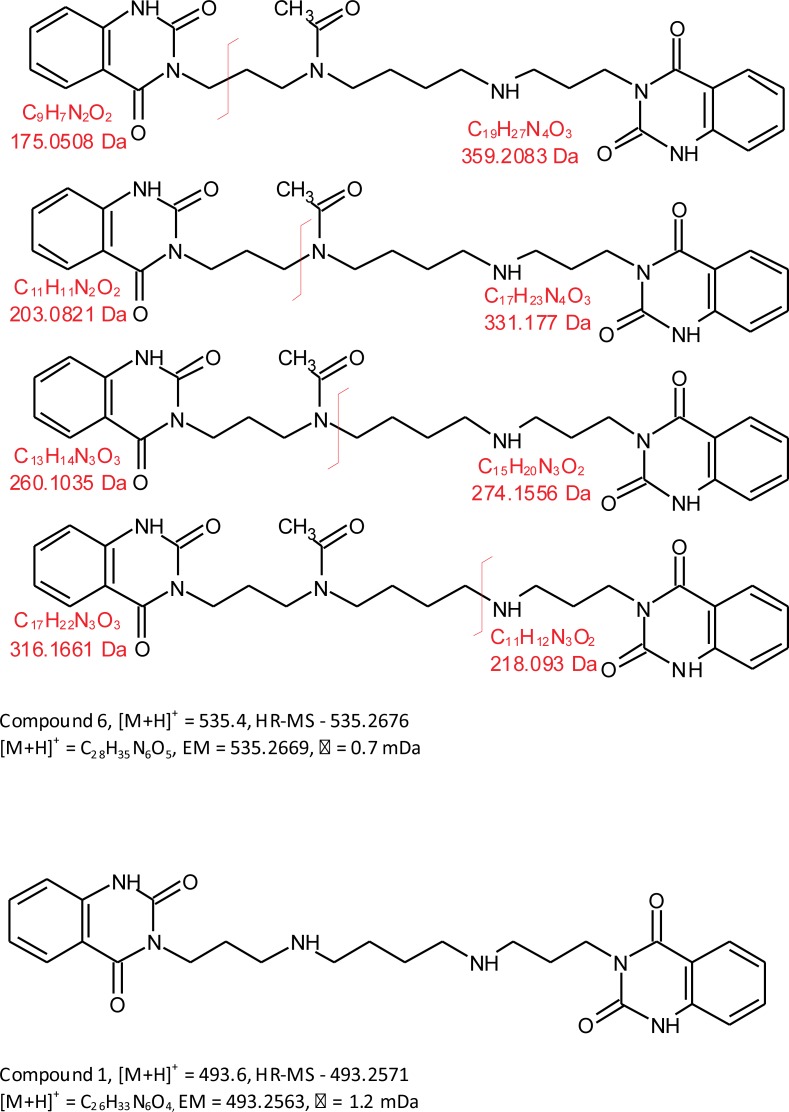
The structure of the investigated compounds and possible MS-fragmentation pattern of compound 6.

The results of NMR analysis of compound 6 (S1–S5 Figs) led to the structure shown in Fig 4. This structure is consistent with the HR-MS data that gave *m/z* = 535.2676, leading to the formula C_28_H_35_N_6_O_5_ for the [M+H]^+^ ion (EM = 535.2669, Δ = 0.7 mDa). This metabolite is identical to SP-8203 reported earlier in coelomic fluid of *E*. *andrei* [11, 13].

To verify the fluorescence properties of the quinazoline-2,4-dione ring, synthetic *n*-butyl quinazoline-2,4-dione was prepared and its fluorescence properties turned out to be identical to those of compounds 1, 3, 4 and 6 (Table 1, Figs 2 and 3), confirming the structure of the fluorescing moiety of these molecules.

The fragmentation low-resolution (LR) mass spectra of compound 6 (S6 Fig) gave the following ions with *m/z* = 146.2, 175.3, 203.4, 274.4, and 316.5 that correspond to the depicted fragments in Fig 4.

The LC-MS analysis of compound 1, gave [M+H]^+^ = 493.6 and fragments with *m/z* = 146.3, 175.3, 203.3, 247.5, 274.5 and 291.5. As compared to fragments of compound 6, no peak at *m/z* = 316.5 was observed in this case (S6 Fig), suggesting the absence of N-acyl group in the molecule of compound 1 (Fig 4). The proposed structure is consistent with HR-MS [M+H]^+^ data: *m/z* = 493.2571, the formula C_26_H_33_N_6_O_4_ for the [M+H]^+^ ion (EM = 493.2563, Δ = 0.8 mDa).

The LC-MS analysis of compound 3, resulted in [M+H]^+^ of 510.6 and fragments with *m/z* = 121.0, 146.2, 175.4, 178.4, 203.4 and 274.5 (S6 Fig). Also in this case, no peak at *m/z* = 316.5 was found. The HR-MS [M+H]^+^ data: *m/z* = 510.2704, suggest the formula C_27_H_36_N_5_O_5_ for the [M+H]^+^ ion (EM = 510.2716, Δ = 1.2 mDa) which is consistent with the compound suggested to be specific for *E*. *fetida*, containing one quinazoline-2,4-dione ring and the other salicylamide ring [13]. The peak at *m/z* = 178.4 (EM = 178.0868, C_10_H_12_O_2_N) would correspond to the fragment containing the salicylamide ring and three CH_2_ groups [13].

## Discussion

Fluorescence spectra measured on lysates of coelomic fluid retrieved from *E*. *andrei*, most hybrids between *E*. *andrei* and *E*. *fetida*, and a few specimens of the latter species [4–6, 8] are similar to those of compounds 1, 3, 4, and 6 (Fig 3).

The fluorophore corresponding to peak 6, dominating in coelomic fluid of *E*. *andrei* is identical to that described previously and called SP-8203 [11], which was also synthesized and tested pharmacologically [11, 12, 15]. This metabolite has also been independently found in coelomic fluid of *E*. *andrei* [13], although in none of these previous studies its fluorescent properties were reported. In the present study, we have identified also two new compounds for *E*. *andrei*, one corresponding to peak 1 in the HPLC chromatogram, which differs from the compound 6 by the lack of N-acetyl group and was not reported yet in any organisms, as well as a compound that was already identified in *E*. *fetida* [13] and regarded as species specific. However our studies show that this compound occurs also in *E*. *andrei* in significant amounts.

The presented data indicate that in the coelomic fluid of *E*. *andrei* there are also other compounds sharing the same (compound 4) or similar (compounds 2 and 5) chromophore structure with compound 6. These compounds require further studies.

The question remains as to the metabolic origin and physiological function of the investigated compounds. These are most probably formed by condensation of spermine with quinozoline-2,4-dione by earthworms. The first potential substrate was identified in coelomic fluid of *E*. *fetida* [16], while quinazoline derivatives are widespread in nature, especially in plants [17, 18]. To be considered is also bacterial origin of the investigated compounds as almost all lumbricid earthworms harbor extracellular vertically transmitted species-specific bacterial symbionts of the genus *Verminephrobacter* localized in their excetory nephridia [19, 20]. The other question is about physiological function of these compounds in earthworms, which could be antimicrobial and cytotoxic. It is known that quinazolinones have broad pharmacological and biological activity, such as herbicide action, antioxidant, antimicrobial, anticancer, antitubercular activity and others [14, 21].

Riboflavin (vitamin B_2_), having fluorescence spectra quite different from those of presently investigated fluorophores, was originally identified in *E*. *fetida* [22] and then detected in coelomic fluid and/or chloragogenous tissue of several lumbricid species [23, 24]. This vitamin plays a pivotal role in immunity [24–27] and regenerative processes [28]. After experimental expulsion of colomocyte-containing coelomic fluid, both coelomocytes (amoebocytes and chloragocyte-derived eleocytes) and riboflavin are almost completely lost and amount of riboflavin is restored slowly, during 5–7 weeks, in parallel with numbers of chloragocyte-derived eleocytes. In contrast, during such treatment, the UV-emitting fluorophores are only partly depleted and restored much faster, during two-three weeks. It indicates that, in contrast to riboflavin, eleocytes are not the main source of the presently investigated compounds, and might support hypothesis about symbiotic bacterial involvement in metabolic pathways leading to formation of *E*. *andrei*-specific fluorophore.

## Supporting information

S1 Fig Chemical shifts of ^13^C and ^1^H in NMR spectrum of compound 6 isolated from *E*. *andrei* coelomic fluid. (DOCX)

S2 Fig COSY spectra of compound 6 in the aliphatic region. (PDF)

S3 Fig COSY spectra of compound 6 in the aromatic region. (PDF)

S4 Fig Representative ^1^H spectra of compound 6. (PDF)

S5 Fig Representative ^13^C spectra of compound 6. (PDF)

S6 Fig MS fragmentation pattern of compounds 1, 3 and 6. (PDF)
