# Supplementary material for: Identification of new fluorophores in coelomic fluid of Eisenia andrei earthworms
Source: PLoS One. 2019 Mar 28;14(3):e0214757. doi: 10.1371/journal.pone.0214757 (PMC6438515; doi:10.1371/journal.pone.0214757)
Supplement: S1 Fig — (DOCX) [file pone.0214757.s001.docx]

Chemical shifts of ^13^C in NMR spectrum of compound 6 isolated from *E. andrei* coelomic fluid. A and B are rotamers of the molecule, α, β, γ, δ are different conformations of heterocyclic rings.


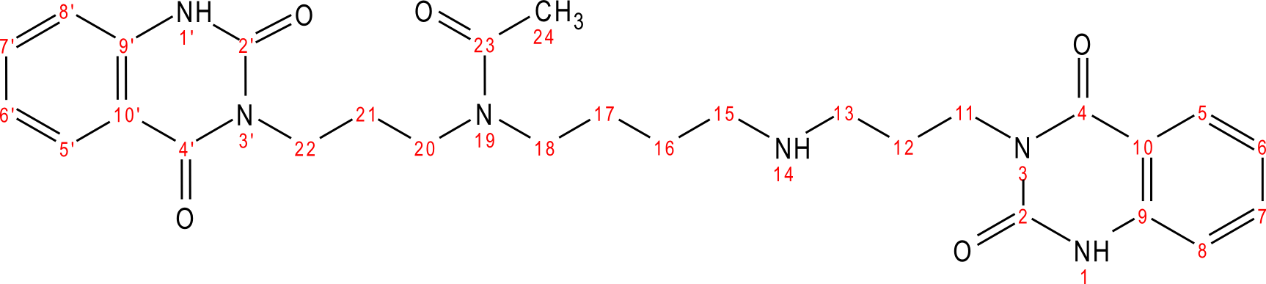


| Chemical shift of ^13^C [ppm] | Atom number | Attribution:  A,B or α, β, γ, δ | Comments |
| --- | --- | --- | --- |
| 173.9161 | 23 | A,B |  |
| 173.9104 |  |  |  |
| 164.4028 | 4’ | A,B |  |
| 164.3776 |  |  |  |
| 163.8226 | 4 | A,B |  |
| 163.8032 |  |  |  |
| 151.5319 | 2’ |  |  |
| 151.5151 |  |  |  |
| 151.4949 | 2 |  |  |
| 151.4806 |  |  |  |
| 138.5512 | 20 | α, β |  |
| 138.5174 |  |  |  |
| 138.3387 | 20 | γ |  |
| 138.2839 | 20 | δ |  |
| 136.0154 | 7 or 7’ | α, β | Superimposition of two signals |
| 135.7719 | 7 or 7’ | γ |  |
| 135.6894 | 7 or 7’ | δ |  |
| 126.8904 | 5 or 5’ |  |  |
| 126.8819 | 5 or 5’ |  |  |
| 126.7553 | 5 or 5’ |  |  |
| 126.6965 | 5 or 5’ |  |  |
| 123.7099 | 6 or 6’ | α, β |  |
| 123.7046 |  |  |  |
| 123.5816 | 6 or 6’ | γ |  |
| 123.5058 | 6 or 6’ | δ |  |
| 115.4640 | 8 or 8’ | α,β |  |
| 115.4466 |  |  |  |
| 115.1520 | 8 or 8’ | γ, δ |  |
| 115.0816 |  |  |  |
| 113.1749 | 10 or 10’ | γ, δ | Superimposition of two signals |
| 113.0630 | 10 or 10’ | α, β |  |
| 113.0117 |  |  |  |
| 49.3599 | 18 | 12B |  |
| 47.6856 | 20 | 11A |  |
| 47.2504 | 15 | 15B |  |
| 47.2054 | 15 | 15A |  |
| 46.1406 | 18 | 12A |  |
| 44.7649 | 20 | 11B |  |
| 44.7154 | 13 | 16A,B |  |
| 44.6897 |  |  |  |
| 38.9646 | 22 | 9B |  |
| 38.6308 | 22 | 9A |  |
| 37.4786 | 11 | 18A,B |  |
| 37.4381 |  |  |  |
| 26.8612 | 21 | 10A |  |
| 26.1911 | 21 | 10B |  |
| 25.4755 | 17 | 13B |  |
| 24.4365 | 17 | 13A |  |
| 24.4149 | 12 | 17 A,B |  |
| 24.3654 |  |  |  |
| 22.9752 | 16 | 14A |  |
| 22.8577 | 16 | 14B |  |
| 20.7114 | 24 | A |  |
| 20.6894 | 24 | B |  |

Chemical shifts of ^1^H in NMR spectrum of compound 6 isolated from *E. andrei* coelomic fluid. A and B are rotamers of the molecule, α, β, γ, δ are different conformations of heterocyclic rings.

| Chemical shift of ^1^H [ppm] | Attribution:  A,B or α, β, γ, δ | Comments |
| --- | --- | --- |
| 1.616 | 16A |  |
| 1.658 | 17A i 16B | Superimposition of two signals |
| 1.745 | 17B |  |
| 1.862 | 21B |  |
| 1.940 | 21A |  |
| 1.953 | 12A and 12B | Superimposition of two signals |
| 2.063 | 24B |  |
| 2.088 | 24A |  |
| 2.936 | 13A and 13B | Superimposition of two signals |
| 2.971 | 15A |  |
| 2.988 | 15B |  |
| 3.329 | 18A |  |
| 3.387 | 20B |  |
| 3.394 | 18B |  |
| 3.438 | 20A |  |
| 3.750 | 22B |  |
| 3.795 | 22A |  |
| 3.888 | 11A and 11B | Superimposition of two signals |
| 6.749 | 8 or 8' δ |  |
| 6.781 | 8 or 8' γ |  |
| 6.892 | 8 or 8' β |  |
| 6.903 | 8 or 8' α |  |
| 7.027 | 6 or 6' δ |  |
| 7.046 | 6 or 6' γ |  |
| 7.103 | 6 or 6' β |  |
| 7.112 | 6 or 6' α |  |
| 7.443 | 7 or 7' γ and δ | Superimposition of two signals |
| 7.529 | 7 or 7' |  |
| 7.535 | 7 or 7' |  |
| 7.573 | 5 or 5' δ |  |
| 7.611 | 5 or 5' β and γ | Superimposition of two signals |
| 7.634 | 5 or 5' α |  |
