# Supplementary figures and images for: Identification of new fluorophores in coelomic fluid of Eisenia andrei earthworms
Source: PLoS One. 2019 Mar 28;14(3):e0214757. doi: 10.1371/journal.pone.0214757 (PMC6438515; doi:10.1371/journal.pone.0214757)

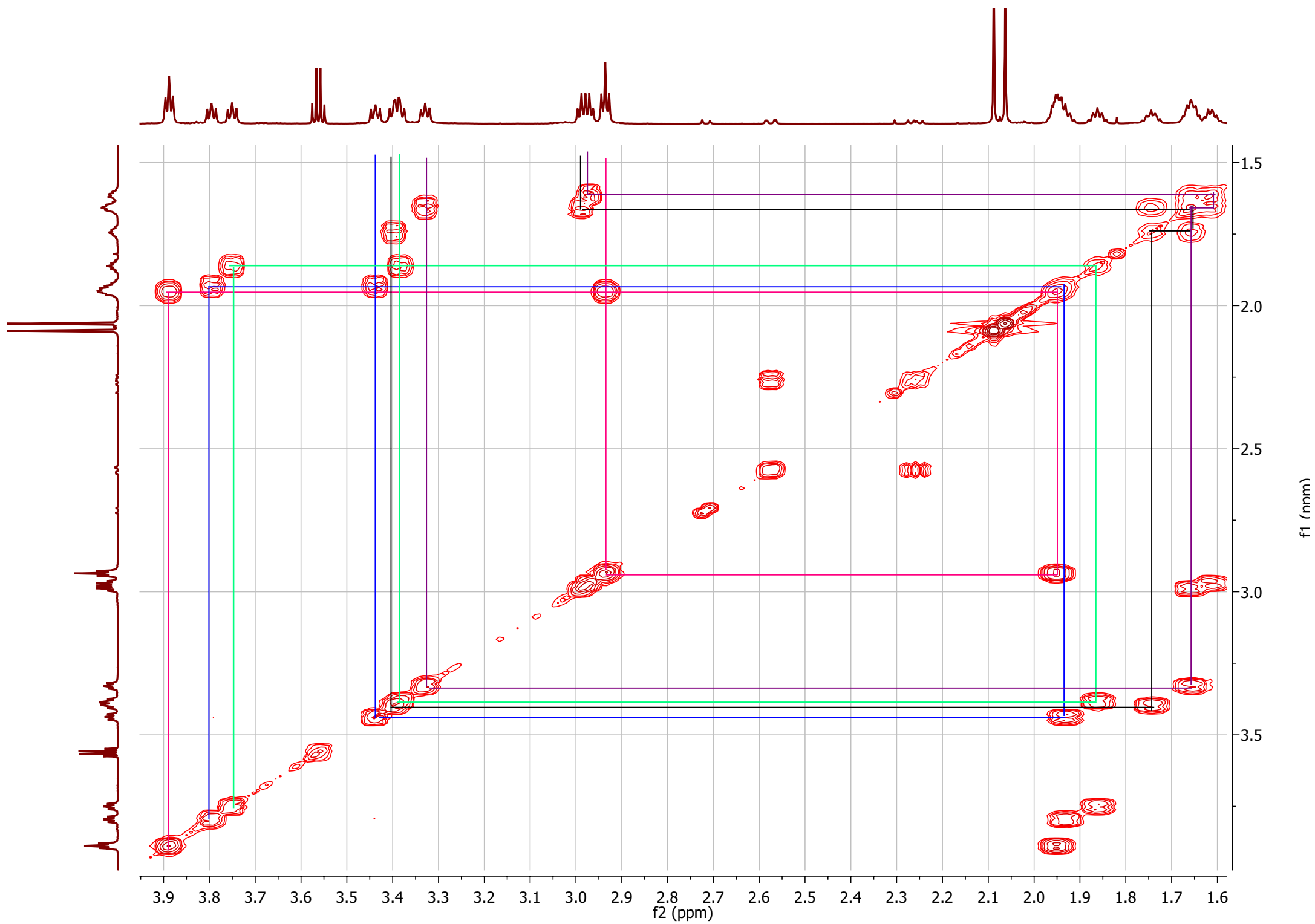

Supplement: S2 Fig — (PDF) [file pone.0214757.s002.pdf]

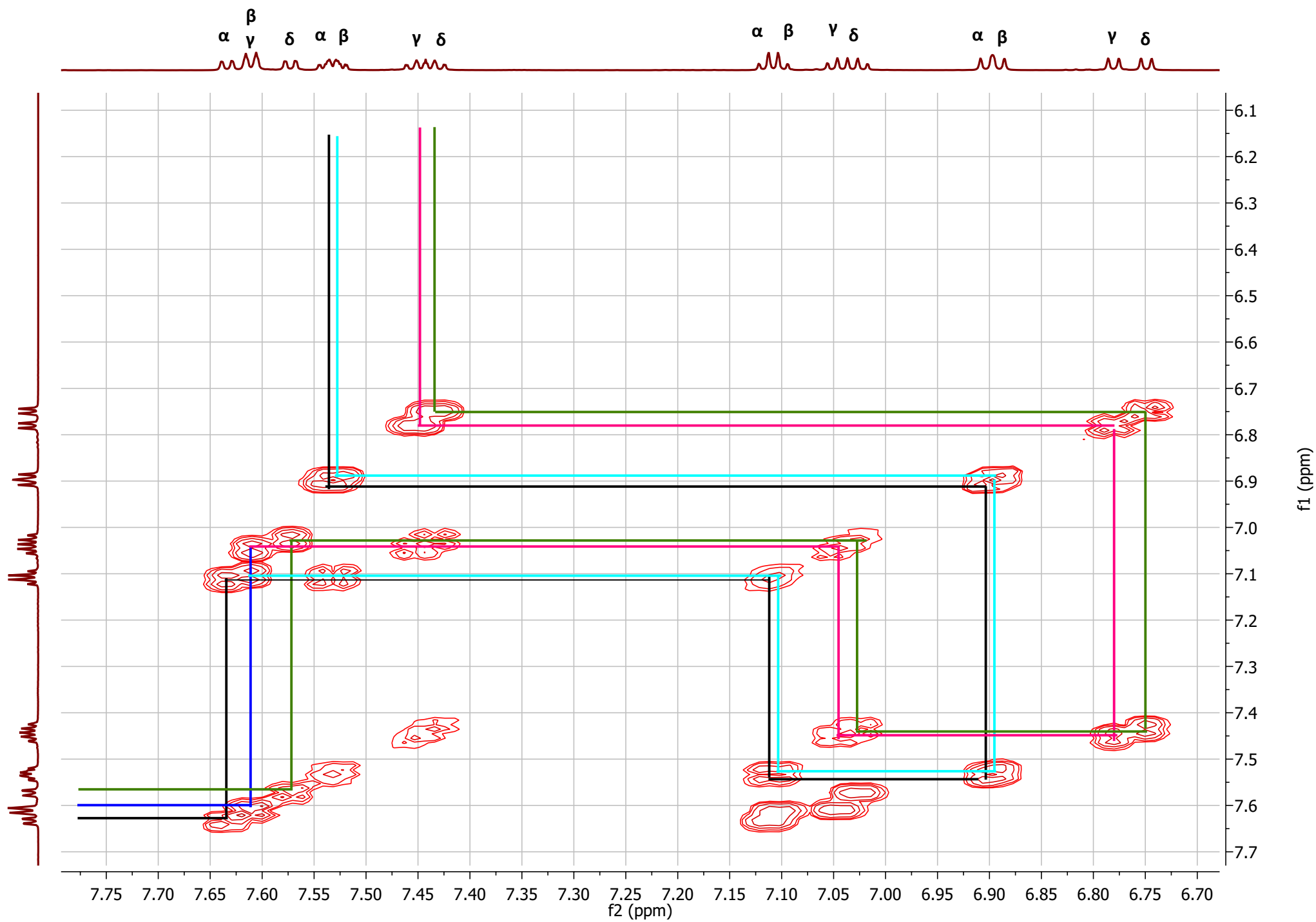

Supplement: S3 Fig — (PDF) [file pone.0214757.s003.pdf]

Water Suppression Spectrum

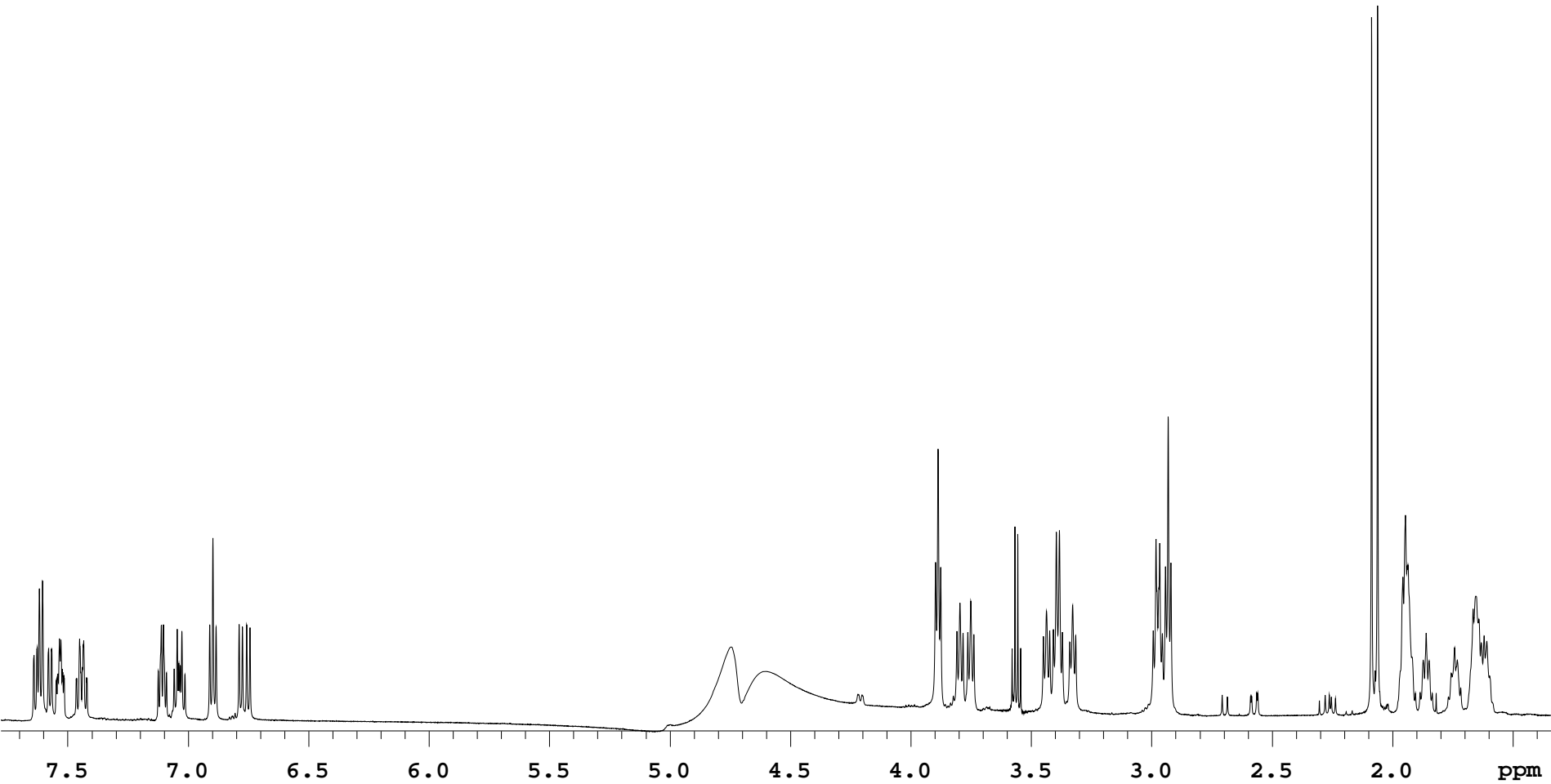

Supplement: S4 Fig — (PDF) [file pone.0214757.s004.pdf]

carbon\_woda 1 1 /acqdata/Kruk

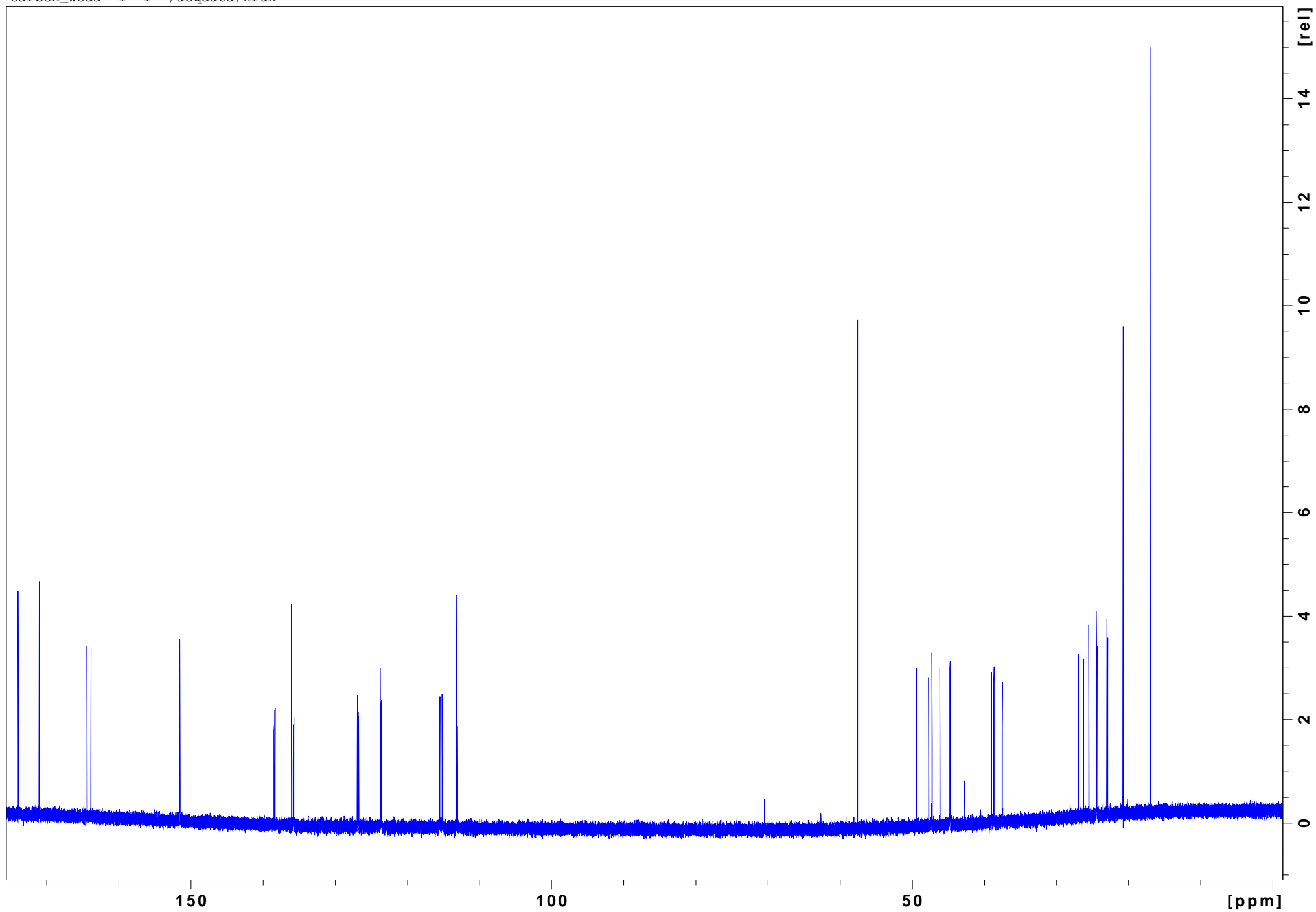

Supplement: S5 Fig — (PDF) [file pone.0214757.s005.pdf]

### Compound 3

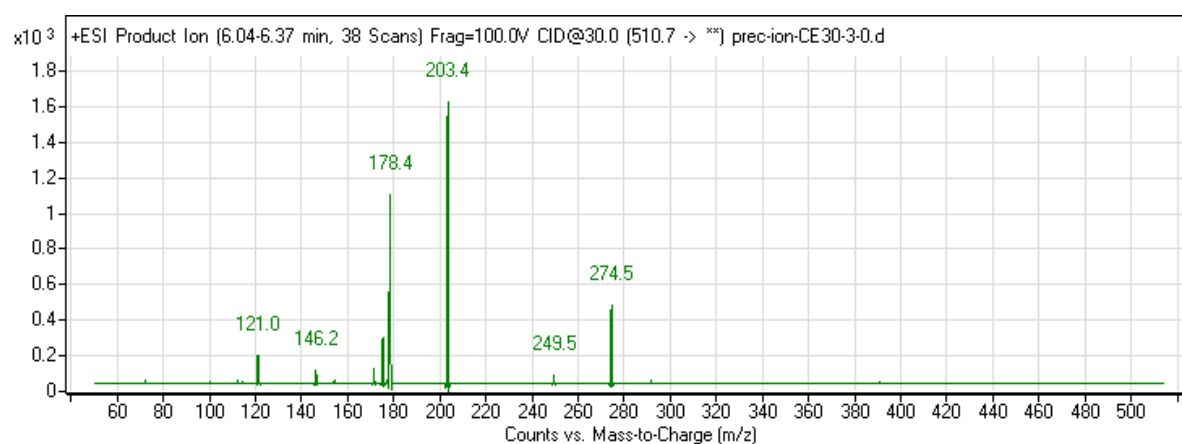

### Compound 1

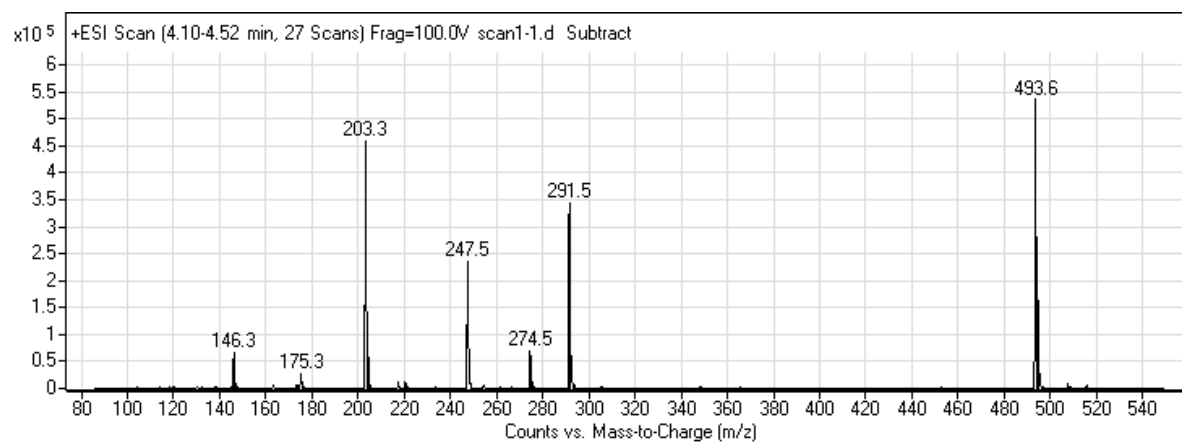

### Compound 6

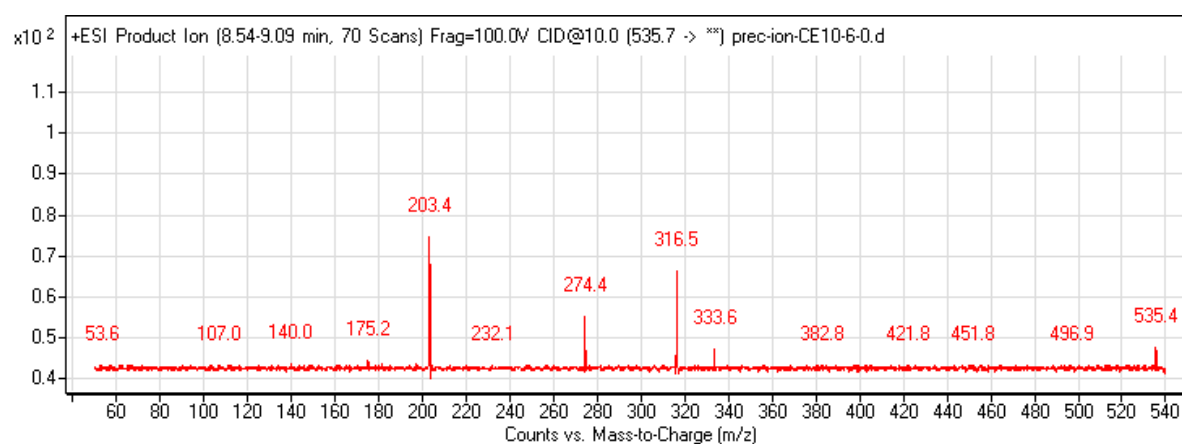

Supplement: S6 Fig — (PDF) [file pone.0214757.s006.pdf]
